# The Effects of the Combination of High-Intensity Interval Training with 3D-Multiple Object Tracking Task on Perceptual-Cognitive Performance: A Randomized Controlled Intervention Trial

**DOI:** 10.3390/ijerph18094862

**Published:** 2021-05-02

**Authors:** Soo-Yong Park, Thomas Jürgen Klotzbier, Nadja Schott

**Affiliations:** Department of Sport and Exercise Science, Institute of Sport Psychology & Human Movement Performance, University of Stuttgart, 70569 Stuttgart, Germany; thomas.klotzbier@inspo.uni-stuttgart.de (T.J.K.); nadja.schott@inspo.uni-stuttgart.de (N.S.)

**Keywords:** 3D-multiple object tracking, cognitive training, perceptual–cognitive skills, working memory, high-intensity interval training

## Abstract

The ability to process goal-related visual information while ignoring goal-irrelevant information is essential for the human attention system. The study aimed to investigate how perceptual–cognitive performance was affected during high-intensity interval training (HIIT) using a 3D-multiple object tracking (3D-MOT) task called Neurotracker (NT). In an experimental design, 42 healthy adults (age *M* = 23.3 *SD* = 2.94, VO_2_max 52.8 ± 5.66 mL·kg^−1^·min^−1^) were randomly assigned to an intervention (HIIT + NT, NT, HIIT) or control group. NT performance (20 trials per session) was measured pre-and post-test (at 5, 15, and 25 min while running on the treadmill). The participants trained twice a week for a 4-week intervention period. There was a significant interaction effect between pre/post-test and groups regarding perceptual-cognitive performance, indicating similar enhancements in the HIIT + NT and the NT group during exercise. HIIT influences physical fitness but did not show any impact on perceptual–cognitive performance. Due to the specific NT task characteristics, improved physical abilities may not directly impact sport-specific perceptual-cognitive performance. Our findings suggest that training resulted in substantial task-specific gains. Therefore, combination training may be proposed as a training program to improve perceptual–cognitive, and physical performance in a time-efficient way.

## 1. Introduction

A flexible allocation of attention resources seems essential (e.g., during a soccer match) to process goal-oriented visual information while selecting irrelevant information. The human attention system must adapt to perceptual–cognitive demands in various sports situations [1]. Perceptual–cognitive performance in sports contexts is considered to play a crucial role for competitive athletes [2]. This is especially true for ball games where athletes are frequently confronted with cognitively demanding situations that require the simultaneous processing of different pieces of information with varying exercise intensities and durations [3]. Intermittent practice with repetitions of short and/or long and high and/or moderate intensities is required to meet the demands of team sports such as soccer, handball, basketball, and hockey.

While perceptual–cognitive skills are typically developed through regular, on-field practice, several training techniques can enhance those skills outside of or in conjunction with regular training (e.g., perceptual–cognitive training (computer projection, Virtual Reality, Quiet eye training, and 3D-MOT task)) [4]. Sports training (and the experience gained through training) is a form of cognitive training that leads to more efficient brain networks and plasticity, leading to cognitive process improvements [2,5,6]. Based on these studies, Faubert and Sidebottom [7] introduced a perceptual–cognitive training program in the form of a 3D-multiple object tracking task called Neurotracker (NT; CogniSens Inc.; Montreal, QC, Canada). NT has been suggested as a cognitive training program to isolate and improve specific cognitive abilities such as selective visual attention or working memory while tracking multiple objects moving in 3D-space. [8]. This training program, which stimulates many brain networks that work together during exercise, including complex motion integration, dynamic, sustained, distributed attention processing, and working memory [9], is of great importance in a dynamic sport context [7]. In most cognitive tasks, response time and correct response rate are assessed for a given number of items at a given fixed speed [10]. Since the NT-system can use the speed threshold as a dependent variable indicating the difference between individuals, the degree of difficulty can be individualized during NT-training, and performance changes can be continuously observed [7]. Previous studies have already demonstrated the training effects of cognitive performance through NT-training, suggesting that optimal processing of sports-related visual scenes and perceptual–cognitive abilities can be trained in athletes [7,11,12,13]. However, perceptual–cognitive performance using NT training has not been studied during physical exercise.

Only a limited number of longitudinal studies evaluate the long-term effects of different exercise interventions on cognitive performance. Most studies show an improvement in various attention components, neurophysiological measures, or both. A recent systematic review by Fernandes et al. [14] indicates that most longitudinal studies are limited to older adults, especially women. Only two studies have been conducted with children [15] and young adult men [16]. While most studies with older adults and children [15,17,18] exhibited positive results, the only study with young adults [16] showed no positive long-term improvements with training during the performance of an attention-demanding task. Fernandes et al. [14] justify this finding by stating that the study by Gutmann et al. [16] used a very low exercise intensity (50% of peak power). Overall, it appears that aerobic exercise, given an adequate training stimulus, is an effective method for improving cognitive performance on both behavioral [17,19,20] and neuroelectric indices [17]. Other potentially effective aerobic training methods, such as HIIT, have not been investigated to the best of our knowledge. Therefore, this study uses a HIIT protocol to examine its effect on cognitive performance.

The current literature shows that the relationship appears to depend on the type of exercise, duration [21], intensity [22,23], physical fitness and demographics [22,24,25], as well as timing and type of cognitive tasks assessed [2,22,23,26]. In the past few years, most studies have been dedicated to examining the effect of different exercise training modalities on cognitive and physical functioning and have measured cognitive performance mainly after exercise and at rest, but rarely during exercise. However, these situations could be of great importance, especially in sports games. Few researchers have studied cognitive performance during a High-Intensity Interval Training (HIIT) protocol, and it is rarely discussed how cognitive performance is influenced during physical exercise. For example, Smith et al. [27] reported that the decision error rate deteriorates during high-intensity exercise. Casanova et al. [27] demonstrated decreased accuracy in a decision-making task due to a prolonged intermittent exercise protocol. HIIT has been shown to produce similar cardiovascular and metabolic health benefits more time-efficient than continuous moderate-intensity exercise. More importantly, it leads to more sustained improvements in prefrontal cortex-dependent executive function during recovery from exercise [28,29]. One of the fundamental mechanisms responsible for high-intensity exercise having a positive effect on cognitive function is the higher brain-derived neurotrophic factor (BDNF) and catecholamine concentrations induced by high-intensity exercise. However, this requires further investigation [30]. It is essential to investigate the longitudinal effects of HIIT on perceptual–cognitive performance to make context-specific and individual recommendations for action concerning exercise load. Thus, we examine the intervention effect of exercise on cognitive performance during physical exercise.

The purpose of this study was to investigate a four-week-intervention effect of HIIT in combination with and without a 3D-multiple object tracking task (NT). Systematic reviews have found that the combination of physical and cognitive training could improve cognition in a more specific manner [31,32]. We thus hypothesized that a combination of HIIT and NT-training would be particularly associated with enhanced long-term effects on perceptual–cognitive performance during physical exercise. This intervention effect may positively impact many team sports by compensating for the decline in cognitive performance during high-intensity exercise.

## 2. Materials and Methods

### 2.1. Participants

Forty-two healthy young adults (M = 23.3, SD = 2.94, age-range 19–30, 13 women) participants volunteered to participate in the experiment. They were recruited from the University Sports Center of the University of Stuttgart, Germany. A questionnaire for sports biography was used to determine what type of sport the participants did and how often they exercised per week to participate in the intervention study. Since sport type determines differences in executive functions in elite athletes [33,34], the sports biography was categorized into static sports (self-paced activities in highly consistent circumstances, e.g., taekwondo, athletics, and dancing, etc.), strategic sports (requiring the adaption to highly varying situations considering teammates, opponents, positions or objects, e.g., soccer, basketball, handball, and volleyball, etc.) or interceptive sports (requiring dynamic coordination between athlete′s body and an implement or environment, e.g., table tennis and tennis, etc.) as well as sport-unspecific training such as strength and endurance training (see [6]). The different categories′ participants were randomly assigned (random number generation function in Excel, [35]) to the three experimental conditions and the control group. All participants received verbal and written information about the study′s nature and aim and signed a consent form. All procedures were in accordance with the Declaration of Helsinki with ethical standards, legal requirements, and international norms. An internal ethics committee of the university approved the study.

### 2.2. Experimental Design and Procedure

To analyze the effects of HIIT on perceptual–cognitive performance during exercise and to examine differences in these changes across interventions, we employed a 4 (intervention: high-intensity interval training + Neurotracker [HIIT + NT] vs. Neurotracker [NT] vs. high-intensity interval training [HIIT] vs. control) × 6 (during HIIT protocol at 5 min, 15 min, and 25 min in pre-and post-test) mixed factorial design. Participants were randomly assigned to one of the four intervention protocols (*n* = 10/11 per intervention group). They were tested twice before and after a 4-week intervention period.

**Figure 1:**
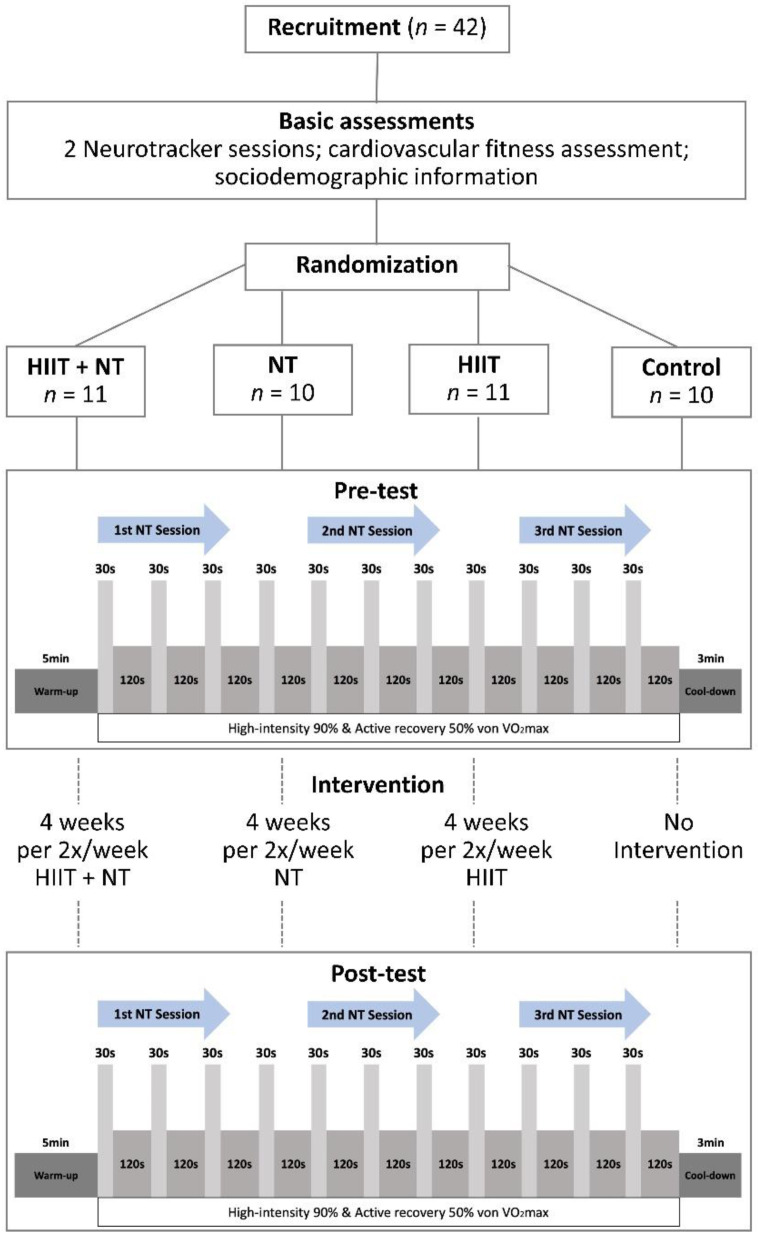
Study design and procedure. The study employed a 4 (four groups) × 6 (5, 15, and 25 min in pre-and post-test) mixed factorial design. HIIT: High-intensity interval training; NT: Neurotracker.

**Baseline assessments:** On the first day, the participants were informed about the study′s procedures and requirements. After that, they gave their written consent and completed the questionnaires. Then, all subjects were asked to perform two sessions (20 trials per session) as a practice version to familiarize themselves with Neurotracker. After a short break, VO_2_max was assessed using the Bruce protocol. Then, participants were randomly divided into four groups (HIIT + NT, NT, HIIT, control). **Pre-test:** Approximately 48 h to 72 h later, the participants returned to the lab and performed the 3D-MOT task (NT) during the individualized HIIT. Each NT assessment lasted approximately 6 min and was performed at 5 min, 15 min, and 25 min during HIIT, respectively. The HIIT protocol was controlled and administered using the h/p/cosmos para control^®^ 4.1.0. Part II of the Mental and Physical State and Trait Energy and Fatigue Scales Questionnaire (MPSTEFS; [36]) was administered before and after testing. **Intervention:** All participants in the active groups (HIIT + NT, NT, HIIT) completed their respective intervention program twice a week, with approximately two days in-between for four weeks in our laboratory. **Post-test:** After the 4-week training program, all participants were reassessed on three NT sessions during HIIT. Again two days later, the cardiovascular fitness assessment (Bruce protocol) was performed (Figure 1).

### 2.3. Intervention

The HIIT treadmill protocol used in the present study consisted of eleven intervals of 30 s at 90% VO_2_max, interspersed by 2 min active recovery periods at 50% VO_2_max. The duration of each HIIT session was approximately 30 min, not including the warm-up phase (5 min) and the cool-down phase (3 min). The participants of the HIIT group performed only the HIIT protocol. The HIIT + NT group performed three NT-sessions with 20 trials at three-time points (at 5 min, 15 min, and 25 min) during the HIIT protocol. The NT group also completed three NT-sessions with 20 trials and similar breaks between the NT-sessions as the HIIT + NT experimental group. The control group received no treatment (Figure 1).

### 2.4. Demographic Information, BMI, Physical Activity, and Mental/Physical Fatigue

Participants′ demographic information was recorded, participants′ height and weight were measured, and body mass index (BMI, kg/m^2^) was calculated. Additionally, the questionnaire recorded the type of sport practiced by the participants in sports clubs and the number of training sessions they executed per week, the duration of the training sessions, the level of athletic performance, the age at which the participants began regular training, and the athletic success achieved in participation in competitions (local, regional, national, or international tournaments). Participants also completed Part II of MPSTEFS [36] to detect changes in perceived feelings of energy and fatigue before and after the pre-and post-test experiment. A total of 12 separate ratings were obtained for Physical Energy, Physical Fatigue, Mental Energy, and Mental Fatigue.

### 2.5. 3D-Multiple Object Tracking Task

The 3D-MOT (3D Multiple Object Tracking) is a perceptual–cognitive training program under the Neurotracker (NT) system licensed by the University of Montreal (CogniSens Inc.; Montreal, QC, Canada). It is a commercial equivalent of the 3D multiple object tracking [8] for measuring visual tracking speed (VTS) [12]. The VTS score (i.e., NT score or performance) has proven to be a valid indicator of high-order cognitive function [9,37]. The transfer effect onto the sports field for athletes was also demonstrated [9].

**Figure 2:**
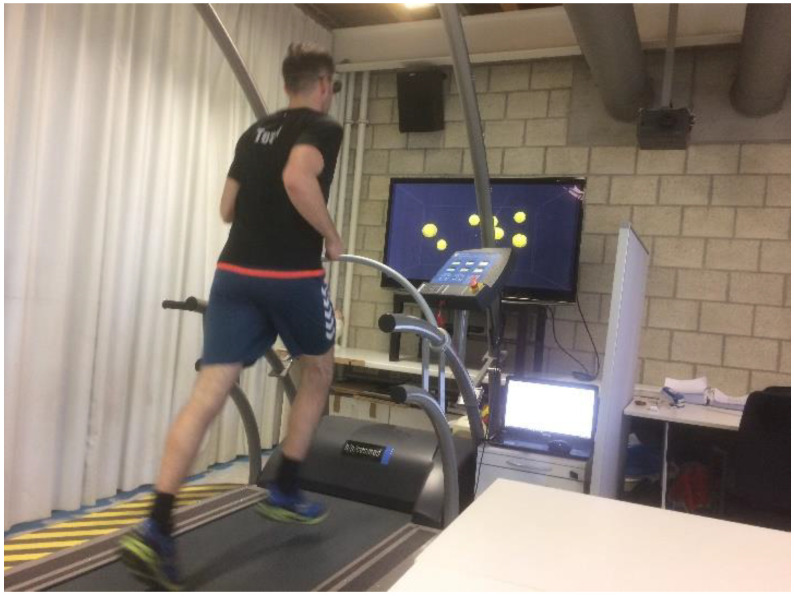
Participant performing the NT task during running on the treadmill.

**Figure 3:**
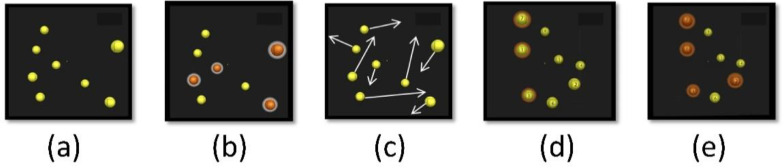
3D-MOT (Multiple object tracking) task. (**a**) Presentation of randomly positioned spheres in a virtual volumetric space; (**b**) Identification of the spheres to be tracked during the trial; (**c**) Removal of identification and movement of all spheres with dynamic interactions; (**d**) Observer′s response by identifying the spheres; (**e**) Feedback is given to the observer Reprinted with permission from ref. [8]. Copyright 2013 Jocelyn Faubert.

Participants stood or ran on a treadmill with 3D-glasses at a 46-degree angle in front of a 3D television (Samsung, 65 inches; Figure 2). In the first phase, eight balls were presented, then four of these eight balls were highlighted in a different color for one second and then returned to their original color. In the next phase, the balls moved at a defined speed in 3D-space for eight seconds. The participant′s task was to track the four initially color-marked balls and then, when they came to a stop, to give a verbal response that identified the four balls illuminated originally at the beginning of the experiment by their number. This was followed by a feedback phase in which the participant named the balls′ numbers, and the subsequent trial began. Participants had to keep their gaze centered and peripherally follow the balls. The five phases of the NT task that occurred within a trial can be seen in Figure 3.

A session consists of 20 trials, lasting a total of approximately 6–7 min. The balls′ speed is adjusted from trial to trial, depending on the accuracy of the response. If all balls are correctly identified, the speed at which the balls move through 3D-space is increased by 0.05 log in the subsequent trial [9,38]. However, if the answers are partially or entirely incorrect, the balls′ speed is reduced by the same proportion, making the task easier. This method is used to calculate a session-specific speed threshold (staircase method; [39]), which is then used as a performance measure (NT performance). The speed at which a new session starts is also adjusted to the performance of the previous session. Overall, this results in a highly adaptive and individualized procedure within and across sessions. VTS has been defined as the fastest speed (in centimeters per second) at which the participant can correctly identify all four illuminated balls with 100% accuracy [40,41].

### 2.6. Maximum Oxygen Uptake (VO_2_max) and Running Speed

In the present study, the Bruce protocol [42] was performed on a treadmill (model: h/p/cosmos pulsar^®^ 3p, Nussdorf-Traunstein, Germany) to determine maximum oxygen uptake (VO_2_max). The Bruce protocol starts with an initial speed of 2.7 km·h^−1^ and an incline of 10%. Every 3 min, the incline increases by 2% per level, and the speed increases by 1.3 km·h^−1^ per level. This testing protocol was terminated when a participant could no longer run due to fatigue. Heart rate (HR) was measured during the protocol using a Polar H1 Heart Rate Sensor (Polar Electro Europe AG, Steinhausen, Switzerland). Maximum HR (HRmax) was determined at the end of the protocol. Thus, an individualized training protocol was applied for all participants in the present study.

### 2.7. Data Analysis

Statistical analyses were implemented on SPSS v.27 (SPSS, Chicago, IL, USA). We first explored dependent variables to examine missing data points, normality of distributions (tested by Kolmogorov–Smirnov tests), homogeneity of variance using Levene′s tests, and outliers′ presence. An alpha level of 0.05 was used for all statistical tests. Characteristics of the study population: potential group differences for continuous variables (i.e., age, height, weight, BMI, physical activity) were assessed using ANOVAs, and categorical demographic variables (i.e., sex) were compared by chi-square test. For ANOVAs, the partial eta2 (η_p_^2^) was calculated as an effect strength measure. Repeated measures sphericity issues were addressed with the Greenhouse Geisser correction. When ANCOVAs were statistically significant, post hoc comparisons were performed using the Bonferroni correction. The classification of the η_p_^2^ follows the conventions of Cohen [43]: 0.01 small effect; 0.06 medium effect; 0.14 strong effect.

**Physical and mental energy/fatigue:** Concerning the MPSTEFS, a 2 (pre-/post-exercise) × 4 (group) repeated measures ANCOVA controlled for sex and HRmax was used for all variables by calculating the rate of change between pre-and post-exercise (physical and mental energy/fatigue). **Maximum oxygen uptake (VO_2_max):** In relation to post-intervention changes in VO_2_max, a 2 (time: basic assessments and post-test) × 4 (group) repeated measures ANCOVA, controlled for sex and VO_2_max of basic assessments was applied.

**Effects of HIIT on the NT performance in pre-test:** A 3 (time: NT score during exercise at 5, 15, and 25 min) × 4 (group) repeated measures ANCOVA, controlled for sex VO_2_max of basic assessments was performed. **NT performance during the intervention period:** A 24 (time: all NT score of eight training sessions) × 2 (group) repeated measures ANCOVA, controlled for sex and the percentage change of VO_2_max was applied to compare the differences of NT performance in the HIIT + NT and NT group during the intervention period. A coefficient of determination (R^2^) of HIIT + NT and NT group was calculated by linear regression between the NT performance and the intervention within four weeks. **Long-term effects on NT performance during HIIT after Intervention:** A 3 (time: NT score during exercise at 5, 15, and 25 min) × 2 (pre-/post-exercise) × 4 (group) repeated measures ANCOVA, controlled for sex and the percentage change of VO_2_max was used. A 3 (time: the percentage change of NT at 5, 15, and 25 min) × 4 (group) ANCOVA with repeated measures controlled for sex and the percentage change of VO_2_max was performed. For the repeated measures ANCOVA used above, the within-subjects factor time and pre-/post-exercise were used, and the between-subjects factor was used with the group.

## 3. Results

### 3.1. Characteristics of the Study Population

Table 1 summarizes the mean age, height, weight, BMI, and physical activity duration by group. At baseline, there were no significant differences between the four groups regarding demographic information. Homogeneity by Levene′s tests was observed equally for the data of participant′s characteristics.

### 3.2. Change Rate of MPSTEFS in Pre-and Post-Test

For the rate of change of all variables (physical and mental energy/fatigue), a 2 (pre-/post-exercise) × 4 (group) repeated measures ANCOVA, controlled for sex and HRmax, showed no significant effect between pre-and post-test. Levene′s tests also indicated equal variances for each variable.

### 3.3. Maximum Oxygen Uptake (VO_2_max)

A 2 (time: basic assessments and after post-test) × 4 (group) repeated measures ANCOVA, controlled for sex and VO_2_max of basic assessments revealed a significant interaction between time and group, *F*(3,36) = 6.04, *p* = 0.002, η_p_^2^ = 0.335, indicating increases in VO_2_max only for the HIIT and the HIIT + NT groups (Table 2). Post hoc analysis demonstrated that the HIIT group significantly differed from NT (*p* = 0.02) and the control group (*p* = 0.07).

### 3.4. NT Performance during HIIT in Pre-Test

**Figure 4:**
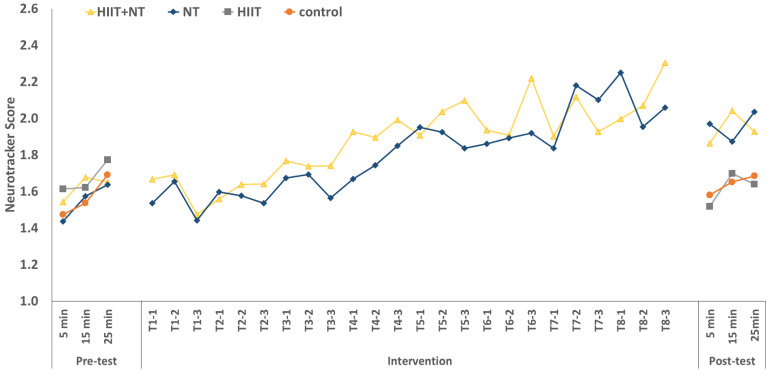
Neurotracker scores over time stratified by group (T1–T8: Training units).

It can be seen that during the HIIT protocol, NT scores increase with increasing time (at 5, 15, and 25 min) in all participants (Figure 4). A 3 (time: during exercise at 5, 15 and 25 min) × 4 (groups) repeated measures ANCOVA, controlled for sex and VO_2_max of baseline assessments revealed a significant effect of time, *F*(2,74) = 3.33, *p* = 0.041, η_p_^2^ = 0.082. No significant interaction was found between time and groups. Post hoc analysis found a significant difference between 5 min and 25 min for the NT Score for all participants (*p* = 0.01).

### 3.5. Long-Term Effects on NT Performance during HIIT after Intervention

**Figure 5:**
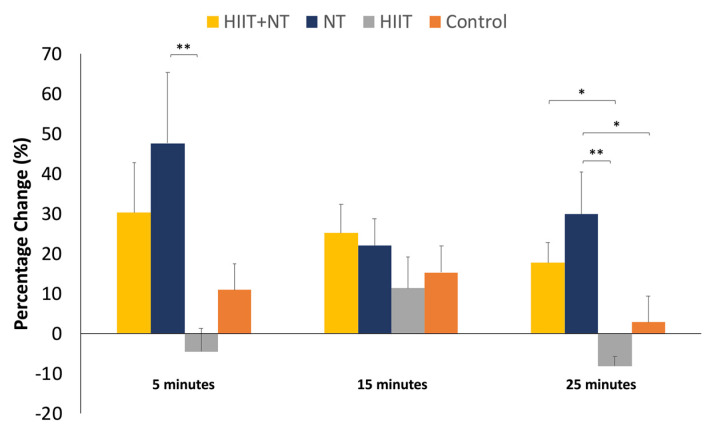
Percentage change of NT scores between pre-and post-test stratified by group. * *p* < 0.05; ** *p* < 0.01.

A 3 (time; during exercise at 5, 15 and 25 min) × 2 (pre-/post-exercise) × 4 (group) repeated measures ANCOVA, controlled for sex and the percentage change of VO_2_max revealed for the NT score a significant effect of pre-/post-exercise, *F*(1,37) = 15.5, *p* < 0.001, η_p_^2^ = 0.296, and interaction between pre-/post-exercise and group, *F*(3,37) = 8.37, *p* < 0.001, η_p_^2^ = 0.404. In addition, there was a significant effect of time, *F*(2,74) = 4.62, *p* = 0.013, η_p_^2^ = 0.111. For the percentage change of NT score, a 3 (time: at 5, 15, and 25 min) × 4 (group) repeated measures ANCOVA controlled for sex and the percentage change of VO_2_max did not demonstrate any significant value, but significant differences were found between groups. At 5 min, there was only a significant difference between NT and HIIT (*p* = 0.004), and at 15 min, no significant difference was found. Finally, as presented in Figure 5, at 25 min, a significant difference was observed between HIIT and HIIT + NT (*p* = 0.047), and the NT group was significantly different from HIIT (*p* = 0.002) and control group (*p* = 0.048).

## 4. Discussion

The present study aimed to examine the effect of HIIT in combination with and without a 3D-MOT (NT) training on the development of perceptual–cognitive performance after a four-week intervention period. We hypothesized that the combination of HIIT and NT-training would enhance the long-term effects on perceptual–cognitive performance during physical exercise. It is well known that exercise has positive effects on cognition [44]. However, few studies investigate the effects of combined physical and cognitive training on perceptual–cognitive performance during exercise. Our study examined the effects of interventions on perceptual–cognitive performance during the HIIT protocol, focusing on long-term effects during physical exercise in young adults.

In the present study, HIIT combined with NT training improved perceptual–cognitive performance. However, a significant improvement of perceptual–cognitive performance during the HIIT protocol was not found. As expected, an improvement was observed during the NT intervention, showing that NT training itself has a high training effect irrespective of the intervention condition.

Amateur athletes′ cognitive abilities reached a certain level with increasing intensity and decreased at the high-intensity level [3]. In contrast, professional athletes′ performance increased despite the high-intensity, which seems to contradict the inverted U-theory [45]. This shows a dependency on the fitness level of the participants [22]. Notably, there were no differences between the groups in terms of NT performance in the pre-test in the present study. This may be attributed to the fact that all participants were having similar VO_2_max levels and high fitness levels according to the VO_2_max normative data (Heywood [46] ((Table 2). Additionally, no significant changes were found at the subjective level for physical and mental fatigue. For this reason, the HIIT protocol used in the present study may not induce fatigue and may have resulted in optimal arousal levels.

Our study examined perceptual–cognitive performance using the NT task during a HIIT protocol for the first time. It showed a high increase in NT performance for the intervention period under two conditions while standing and exercising/running on the treadmill. We observed a significant time effect on perceptual–cognitive performance improvement during the intervention period (*p* < 0.001), but no group differences were found, indicating a high training effect of NT on performance in both groups (HIIT + NT: R^2^ = 0.73; NT: R^2^ = 0.80). Similarly, Faubert and Sidebottom [7] examined perceptual–cognitive performance measured with the NT task under different conditions (sitting vs. standing) with 30 NT sessions. Their results demonstrated that performance during standing was not so dramatically increased compared to performance during sitting, which can be attributed to the relationship between postural control mechanisms and perceptual–cognitive demands.

### 4.1. The Importance of Combining Physical Exercise with Perceptual–Cognitive Training

Many studies suggested that either physical exercise [44] or cognitive training [47,48] has positive effects on the brain′s functional or structural changes. Physical exercise can also strongly stimulate brain function [44,49,50], and its positive effects can potentially be enhanced with combined cognitive training [51]. Combining physical exercise with cognitive tasks may be particularly effective in producing positive, supportive influences on brain structure and function [52]. Nevertheless, the effect of combining two types of intervention is largely unexplored. Few studies have examined the combined effects of exercise and cognitive training, and these have been limited to older adults [31,53,54] or patients with neurological impairment [55]. One study found that specific cognitive training effects improving cognitive function for spatial learning were more pronounced than the effects of physical training [56]. These findings provide evidence that physical exercise alone may not be sufficient to produce improvement in specific cognitive abilities.

For this reason, it could be suggested that specific cognitive training targets relevant functional networks more precisely than a nonspecific physical exercise intervention [32]. In this regard, the significant improvement of NT scores during HIIT protocol was observed only in the HIIT + NT and NT groups in the post-test (interaction between pre-and post-test groups, *p* < 0.001). No improvement in performance was observed in the HIIT and control groups. However, it is somewhat surprising that four weeks of HIIT did not improve perceptual–cognitive performance in the HIIT group. A possible reason for the absence of an effect for HIIT may be due to the short intervention period. Griffin et al. [57] demonstrated that the improvement in cognitive functions concerning memory performance was observed after five weeks of 60 min training at an intensity of 60% of VO_2_max on a cycle ergometer, but not after three weeks. For this reason, it should be investigated whether extending the intervention duration can improve perceptual–cognitive performance in the HIIT group.

Moreover, no studies examine cognitive abilities during physical exercise after interventions, while many studies have investigated cognitive performance at rest after an intervention [14]. However, specific sport-related cognitive training may be required to improve performance during exercise. Interestingly, a similar enhancement in perceptual–cognitive abilities was observed with cognitive training alone compared to the combination of HIIT with NT training. The NT training′s continuous cognitive stimulation during the intervention period could improve the more specific perceptual–cognitive function required for the 3D-MOT task better than the HIIT intervention. Physical exercise could contribute to an overall facilitating effect on learning, such as increased angiogenesis and the availability of specific neurotrophins, which are prerequisites for neuroplasticity [32,51]. Physical exercise can prepare the brain to respond well to cognitive stimulation [58], and cognitive training induces neuronal changes in a specific brain network related to the trained skills [32]. Suppose the combination of physical exercise and cognitive training is continued. In that case, the improvement in task-related cognitive performance could be more significant than if only the cognitive task were trained. Of course, this is just an assumption as we did not measure neural correlates. However, there is a relationship between exercise and cognition on behavioral as well as neural levels [32].

Furthermore, as a mediator that alters brain structure and function, physical exercise could eventually lead to cognitive performance improvements at the behavioral level [58]. However, in the present study, we only examined behavioral data and not neural correlates. Therefore, studies on the NT task′s neural correlates during physical exercise in participants with different fitness levels are needed. It is also necessary to identify the differences between the groups after the intervention period using different cognitive tasks and the NT task (transfer task) and extending the intervention period.

We would like to mention some limitations. First, the relatively small number of participants should be noted. No a-priori power analysis was performed. The primary outcome measure (the Neurotracker Score) was used as part of the two experimental groups′ interventions. The learning effect of the task used for training and assessment makes it difficult to determine whether the improvements were due to training or learning. It is also unclear how this would translate to on-field performance as no transfer test was conducted. Given the short duration of the intervention and the high fitness level at baseline, it is unclear whether the intervention would have affected cognitive performance as a function of improved fitness. HIIT could have a very different physiological effect than high-intensity steady-state exercise (although high intensity), so this must be considered when interpreting study results on high-intensity exercise. For example, in a 30 s HIIT protocol such as this, cerebral blood flow would likely increase in the first 3 s–4 s before decreasing to a level nearly equivalent to rest over the following 25 s. We would expect blood flow velocity to increase and peak during recovery, but we would also expect blood flow to generally decreasing throughout the training session. In short, HIIT affects the brain very uniquely, and this needs to be considered in future studies.

### 4.2. Implications for Future Research and Practice

The combination of physical and cognitive training (as in the HIIT + NT group) can be proposed as an athletic training program to improve overall cognitive and physical performance in a time-efficient way. In particular, a concept of intermittent training or high-intensity interval training for certain team sports and the study of specific perceptual–cognitive performance in such situations would be valuable for all athletes who can train both physical and cognitive skills during real sports.

## 5. Conclusions

In conclusion, perceptual–cognitive training may play a crucial role in improving an athlete′s ability by using sports-specific visual information to facilitate sports-specific skills such as prediction and decision making [4]. The study aimed to investigate how perceptual–cognitive performance was affected during high-intensity interval training (HIIT) using a 3D-multiple object tracking task. Our findings suggest that training resulted in substantial, task-specific gains (physical fitness, perceptual–cognitive performance), but there was no effect of HIIT on perceptual–cognitive performance. Due to the specific 3D-MOT task characteristics, improved physical abilities may not directly affect perceptual–cognitive performance related to sport-specific tasks. The absence of an additional training effect of the HIIT or HIIT + NT compared to the NT group is only somewhat surprising and maybe (a) due to the nature of the HIIT protocol, (b) the short intervention period, and/or (c) the difference between this specific 3D-MOT task and the usually tested cognitive abilities.

## Figures and Tables

**Table 1:** Participant characteristics.

	HIIT + NT (*n* = 11) M (SD)	NT (*n* = 10) M (SD)	HIIT (*n* = 11) M (SD)	Control (*n* = 10) M (SD)	*p*-Value
Sex (male:female)	8:3	6:4	8:3	7:3	0.913
Age (years)	22.9 (2.81)	22.7 (2.79)	24.9 (3.86)	22.6 (1.43)	0.218
Height (cm)	180 (0.70)	175 (0.12)	176 (0.74)	177 (0.10)	0.600
Weight (kg)	73.6 (8.23)	71.7 (14.0)	67.8 (8.27)	70.2 (13.1)	0.662
BMI (kg·m^−2^)	22.7 (1.49)	23.3 (2.16)	21.7 (1.22)	22.10 (2.58)	0.290
Physical activity (min/week)	244 (111)	258 (94.0)	205 (42.0)	171 (69.8)	0.095

M = mean, SD = standard deviation.

**Table 2:** VO_2_max at before pre-test and after post-test stratified by group.

	HIIT + NT (*n* = 11) M (SD)	NT (*n* = 10) M (SD)	HIIT (*n* = 11) M (SD)	Control (*n* = 10) M (SD)	*p*-Value
HRmax (bpm)					
Basic assessments	190 (7.65)	191 (4.84)	189 (8.15)	192 (4.25)	0.833
After post-test	190 (7.31)	190 (4.97)	188 (7.39)	192 (5.51)	0.783
VO_2_max (mL·kg^−1^·min^−1^)					
Basic assessments	53.6 (6.09)	52.2 (4.91)	51.6 (6.76)	53.8 (5.07)	0.644
After post-test	55.7 (5.91)	52.1 (6.06)	54.8 (6.42)	53.0 (4.25)	0.557

M = mean, SD = standard deviation.

## Data Availability

All relevant data are within the study.
